# Dynamics of salivary markers of kidney functions in acute and chronic kidney diseases

**DOI:** 10.1038/s41598-020-78209-1

**Published:** 2020-12-04

**Authors:** Alexandra Gaál Kovalčíková, Kristína Pavlov, Róbert Lipták, Marianna Hladová, Emese Renczés, Peter Boor, Ľudmila Podracká, Katarína Šebeková, Július Hodosy, Ľubomíra Tóthová, Peter Celec

**Affiliations:** 1 Institute of Molecular Biomedicine, Faculty of Medicine, Comenius University, Sasinkova 4, 811 08 Bratislava, Slovakia; 2 Department of Pediatrics, National Institute of Children’s Diseases, Faculty of Medicine, Comenius University, Bratislava, Slovakia; 3 Department of Molecular Oncology, Cancer Research Institute, Biomedical Research Center of Slovak Academy of Sciences, Bratislava, Slovakia; 4 Institute of Physiology, Faculty of Medicine, Comenius University, Bratislava, Slovakia; 5 Institute of Pathology & Department of Nephrology, University Clinic of the RWTH, Aachen, Germany; 6 Institute of Pathophysiology, Faculty of Medicine, Comenius University, Bratislava, Slovakia

**Keywords:** Biochemistry, Biomarkers, Nephrology

## Abstract

Saliva can be used as an alternative diagnostic fluid enabling easy and non-invasive disease monitoring. Urea and creatinine can be measured in saliva and both were shown to be increased in renal failure. However, the dynamics of these markers during the development of kidney diseases is unknown. We aimed to describe the dynamics of salivary urea and creatinine in various animal models of acute kidney injury (AKI) and chronic kidney disease (CKD) and in patients with different stages AKI or CKD. Ninety Wistar rats underwent bilateral nephrectomy (BNX), ischemia–reperfusion injury (IRI) or glycerol-induced kidney injury to model AKI. CKD was modelled using 5/6 nephrectomy. In the clinical part 57 children aged 12.6 ± 4.9 years with AKI (n = 11) or CKD (n = 46) and 29 healthy controls (aged 10.2 ± 3.7 years) were enrolled. Saliva and blood samples were collected in both, animal experiments and the human study. In animal models of AKI, plasma urea and creatinine were higher than in controls. An increase of salivary urea and creatinine (twofold) was observed in BNX and IRI, but only after 12 h and 24 h, respectively. In glycerol nephropathy and 5/6 nephrectomy, salivary urea increased (by 100% and by 50%), while salivary creatinine did not change during the observation period. Salivary urea and creatinine were significantly higher in all patients compared to controls (threefold) and in both, AKI and CKD they were associated with the severity of renal failure. Plasma and salivary concentrations correlated only in children with renal failure (R = 0.72 for urea; R = 0.93 for creatinine), but not in controls (R = -0.007 for urea; R = 0.02 for creatinine). Our study indicates that during the development of renal impairment saliva could be used for non-invasive monitoring in higher stages of AKI or CKD, rather than for screening of early stages of kidney diseases.

## Introduction

Widely used markers for the assessment of kidney functions are creatinine and urea measured in blood plasma^1–3^. Repeated blood sampling needed for monitoring of renal functions is associated with a risk of complications^4^. In combination with haemodialysis, repeated blood collections increase the risk for infections^5^. Using saliva as the diagnostic fluid represents a non-invasive alternative, collection can be conducted at home and does not require trained personnel^6^. These advantages are of special importance for children and elderly patients with comorbidities.

Several clinical and experimental studies have demonstrated increased levels of salivary urea and creatinine in individuals with AKI as well as with CKD in comparison to healthy controls. Moreover, the lowest concentrations were found in patients with early stages of kidney disease which increased with the severity of kidney disease^7–10^. On the other hand, other studies have shown that concentrations of salivary urea and creatinine in patients with early stages of kidney disease (stage 2 and 3) were similar to those in healthy controls. Significantly higher concentrations of salivary urea and creatinine were found only in patients with more severe kidney disease (stage 4 and 5)^8,11^. In some studies, a positive correlation between plasma and salivary concentrations of urea and creatinine has been revealed only in patients with kidney disease, not in healthy controls^7,8,10,12,13^. On the contrary, others have shown a relation between plasma creatinine and urea and their salivary concentrations in both, healthy controls and patients with kidney disease^14,15^. These contradictory results prevent the use of saliva for the assessment of kidney function in the clinics. One of the reasons for the differences between studies may be the different disease severity of the studied patient population. It has been shown that adult and pediatric patients with a higher stage of CKD have also higher salivary markers of renal function^10,11^. In addition, a decrease in salivary urea and creatinine was observed in patients after kidney transplantation and after dialysis^13^.

Previously published studies—either clinical or experimental—focused on single time points^12,16–18^. The largest study published so far focusing on salivary creatinine has shown that serum creatinine can be deduced from its salivary concentrations^18^. However, this was true only for patients with higher stages of CKD. None of the studies analysed the dynamics of salivary markers of renal function during the development of renal diseases. It is also not clear whether saliva can be used for the screening of early stages of kidney diseases.

Our study aimed to evaluate salivary urea and creatinine dynamics in the development and progression of kidney failure using multiple models of AKI and CKD and to confirm these findings in a pediatric cohort of patients with different stages of AKI or CKD.

## Methods

### Animal experiments

Animal experiments were conducted according to the guidelines for laboratory studies performed on animals approved by the Ethics Committee of the Institute of Pathophysiology, Faculty of Medicine, Comenius University, Bratislava, Slovakia. Young adult male Wistar rats were used (n = 90, Anlab, Prague, Czech Republic). Animals were in controlled light/dark cycle room, with ad libitum food and water access, with constant temperature (22 ± 2 °C) and humidity (45–65%).

### Bilateral nephrectomy

After 2 weeks of acclimation period, rats were divided into eight groups. To model AKI, 3 different animal models were used: bilateral nephrectomy—BNX, ischemia reperfusion injury – IRI and glycerol-induced nephropathy. Bilateral nephrectomy was performed in one surgical session as described previously^19^. Animals (n = 11) were anesthetized with ketamine and xylazine (100 mg/kg and 10 mg/kg, i.p., in ratio 3:1). Ophthalmic lubricating ointment was applied to the eyes. The skin was disinfected using betadine. Retroperitoneal incision was made on one side and the renal pedicle was identified. The kidney was decapsulated and renal pedicle was ligated with a suture. The kidney was removed on both sides. The muscle layer was closed using an absorbable suture (Chirlac, HRF 1.5, Chirmax, Praha, Czech Republic). The skin was closed using clips (Sureline Skin Stapler Accessories, 35 W, Patterson Companies, Staffordshire, England, United Kingdom). Control animals (n = 9) underwent the same procedure but the pedicle was not tied off and kidneys were only decapsulated.

### Ischemia–reperfusion injury

To induce IRI (n = 11), rats were anesthetized as mentioned above and surgery was performed as described previously^20^. Ventral approach was used and both kidneys were exposed. In ischemic phase, micro clamps were applied on renal pedicles for 30 min. Kidneys with attached clamps were returned to the abdominal cavity. After 30 min, clamps were released. The muscular layer was closed with an absorbable suture. The skin was closed using clips. The reperfusion phase lasted 48 h. The sham animals (n = 9) underwent the same procedure but pedicles were only gently decapsulated, not clipped.

### Glycerol nephropathy

To induce glycerol-induced nephropathy (n = 11), glycerol (1:1 with saline, 8 ml/kg, Sigma Aldrich, Steinheim, Germany) was applied intramuscularly as described previously ^21^. Briefly, rats in AKI group received single-dose of glycerol equally into both hind limbs. The control group (n = 9) received saline in the same manner.

In all models, blood was collected from the tail vein into microvettes (Microvette 300 Lithium-heparin, microvette 500 EDTA; Sarstedt, Numbrecht, Germany) at baseline and 3, 12, 24 and 48 h after induction of AKI. Blood was centrifuged at 1600 *g* for 10 min. Plasma was stored at − 20 °C until analysis. Prior to saliva collection, rats were anesthetized using ketamine and xylazin as mentioned above. Salivation was induced by intraperitoneal injection of pilocarpine (Unimed Pharma, Bratislava, Slovakia, 0.8 mg/kg of body weight) at same time points as blood collection. Saliva samples were collected using pipettes from the oral cavity into sterile tubes for 30 min.

### 5/6 nephrectomy

To induce CKD, 5/6 nephrectomy (n = 17) was performed in a 2-step surgery^22^. Animals were anesthetized, a retroperitoneal incision was made on the left side and the kidney was exposed. Two thirds of the left kidney were removed. Bleeding was stopped using absorbable gelaspon (Gelita Medical GmbH, Eberbach, Germany). The muscular layer was closed using an absorbable suture. The skin was closed using metal clips. After 2 weeks of recovery, a retroperitoneal incision was made on the right side. The renal pedicle was ligated with a suture, the kidney was decapsulated and removed. In the sham group (n = 13), the kidneys were only decapsulated. Blood was collected from the retroorbital plexus at baseline and after 2, 4 and 6 months after the second surgery. Plasma was stored at – 20 °C until further analysis. Salivation was induced using pilocarpine as described above at the same time points.

### Clinical study

A cross sectional study with age-matched control group was performed. Eighty-one consecutive children with AKI or CKD were enrolled in this study (aged 12.6 ± 4.9). All patients attended the Pediatric nephrology clinic at the National Institute of Children’s diseases, Faculty of Medicine, Comenius University in Bratislava, Slovakia. Patients were enrolled between April 2017 and December 2019. All patients were diagnosed according to Kidney Disease Improving Global Outcomes (KDIGO) guidelines^23,24^. Estimated glomerular filtration rate was calculated according to Schwartz’s equation^25^. Clinical characteristics of the patients are shown in supplementary Table 1. Despite repeated samplings in some patients, each patient was included only once in the study, i.e. after exclusion of samples due to contamination or low volume, samples from 57 patients were further analysed. Children in the control group were enrolled from another study—healthy siblings from the study titled “Markers of oxidative stress in children with Diabetes mellitus type 1” that was approved by the Ethical committee of the National Institute for Children’s Diseases in June 2017. The healthy children were clinically assessed for health status by a pediatrician at the time of blood and saliva collection between June 2017 and December 2019. Any children with signs of chronic or potentially acute disease were excluded from the study. Overall, 98 healthy volunteers participated in the study, however, only 62 healthy children were sampled. Of these, no clinical data were obtained from 13 healthy controls. Of the remaining 49 volunteers, 29 were further enrolled due to age (aged 10.2 ± 3.7) and sex matching. Venous blood was collected into K_3_EDTA tubes (BD Vacutainer Plastic K_3_EDTA Tube, Becton Dickinson, Heidelberg, Germany**)** after overnight fasting in both, healthy controls and patients. Whole unstimulated saliva was collected for 15 min by passive drooling and spitting into sterile collection tubes to a final volume of at least 1 ml (15 ml Tube, Sarstedt, Numbrecht, Germany). To prevent saliva contamination, all participants were asked not to eat, drink or brush the teeth at least 60 min before collection. Health questionnaire assessing general and oral health was filled by the legal guardians. Blood and saliva samples were centrifuged at 1600 *g* for 10 min. The supernatants were stored at − 20 °C until further analysis. Legal guardians of all children have signed written informed consent. This part of the study was conducted according to the principles expressed in the Declaration of Helsinki and according to approvals of the Ethics Committee of the National Institute of Children’s Diseases at the Faculty of Medicine, Comenius University, Bratislava, Slovakia.

### Biochemical analysis

Urea was measured using a commercial kit and spectrophotometric quantification in plasma and saliva (Urea Nitrogen Colorimetric Detection Kit, Arbor Assays, Ann Arbor, USA). For plasma analyses, samples were diluted 1:9, for saliva, samples were diluted 1:1 with distilled water. Absorbance was measured after 30 min of incubation with reagents at room temperature at 450 nm. Limit of detection (LOD) and limit of quantification (LOQ) were 0.05 mmol/l and 0.14 mmol/l respectively. Sensitivity and specificity were calculated as 86.8% and 59.3%, respectively with a cut-off value of 0.15 mmol/l. Area under the receiver operating characteristic (ROC) curve was 0.80 (supplementary Fig. 1). Coefficient of variation (CV) for technical, intra- and inter individual variability were evaluated from samples collected from healthy volunteers repeatedly (supplementary methods). CV of technical, intra- and inter- variability were 10.2%, 44.5% and 49.2%, respectively (supplementary Table 2).

**Figure 1:**
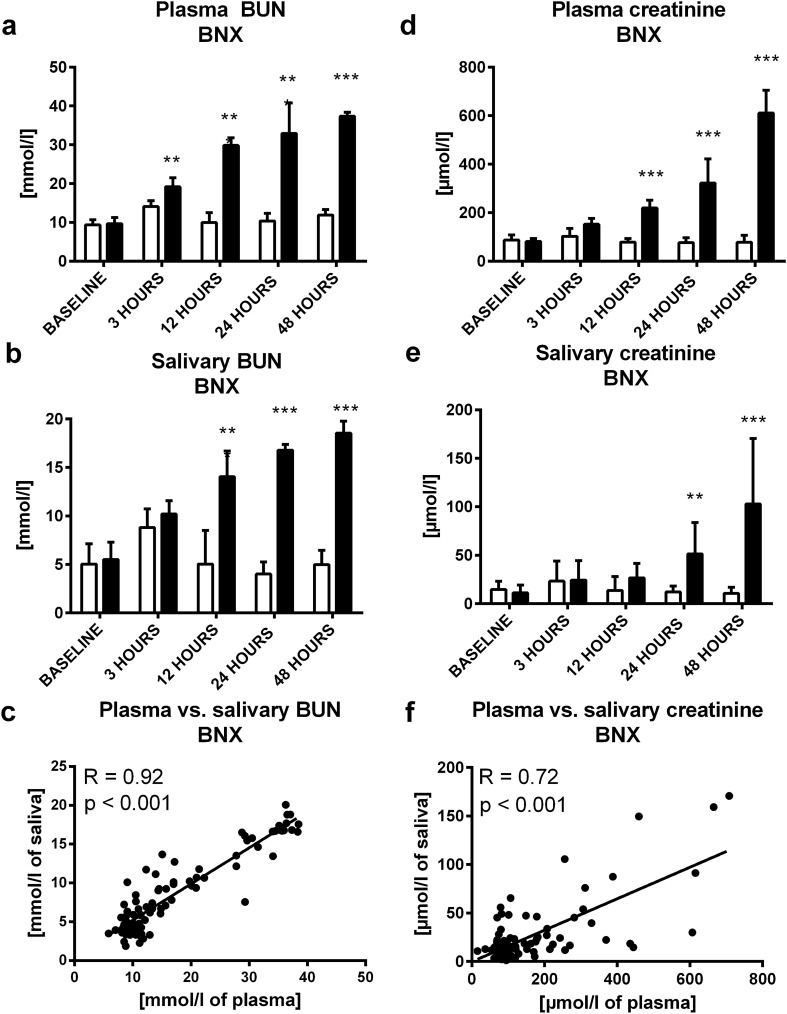
Concentration of urea in BNX or sham group in **(A)** plasma, **(B)** saliva at baseline, 3, 12, 24 and 48 h after bilateral nephrectomy, **(C)** correlation between plasma urea and its salivary concentrations. Concentrations of creatinine in BNX or sham group in **(D)** plasma, **(E)** saliva at baseline, 3, 12, 24 and 48 h after bilateral nephrectomy, **(F)** correlation between plasma creatinine and its salivary concentrations. Sham group is shown as white bars and BNX group as black bars. Results are expressed as mean + SD. ** denotes p ˂ 0.01, *** denotes p˂0.001 in comparison to the sham group (n = 11 for BNX group, n = 9 for control group).

Creatinine in plasma and saliva was measured using a commercial kit (Creatinine Serum Low Sample Volume, Arbor Assays, Ann Arbor, USA) and a spectrophotometric quantification. Briefly, 15 µl of plasma or saliva were transferred into a 384 well plate and absorbance was measured after 1 min and after 30 min of incubation with reagents at 490 nm. LOD and LOQ were calculated as 6.4 µmol/l and 21.1 µmol/l, respectively. Sensitivity and specificity were 87% and 65.5% with cut off value 15.3 µmol/l. Area under ROC curve was 0.80 (supplementary Fig. 2). CV of technical, intra- and inter- variability were 23.2%, 51.8% and 26.9%, respectively (supplementary Table 2).

**Figure 2:**
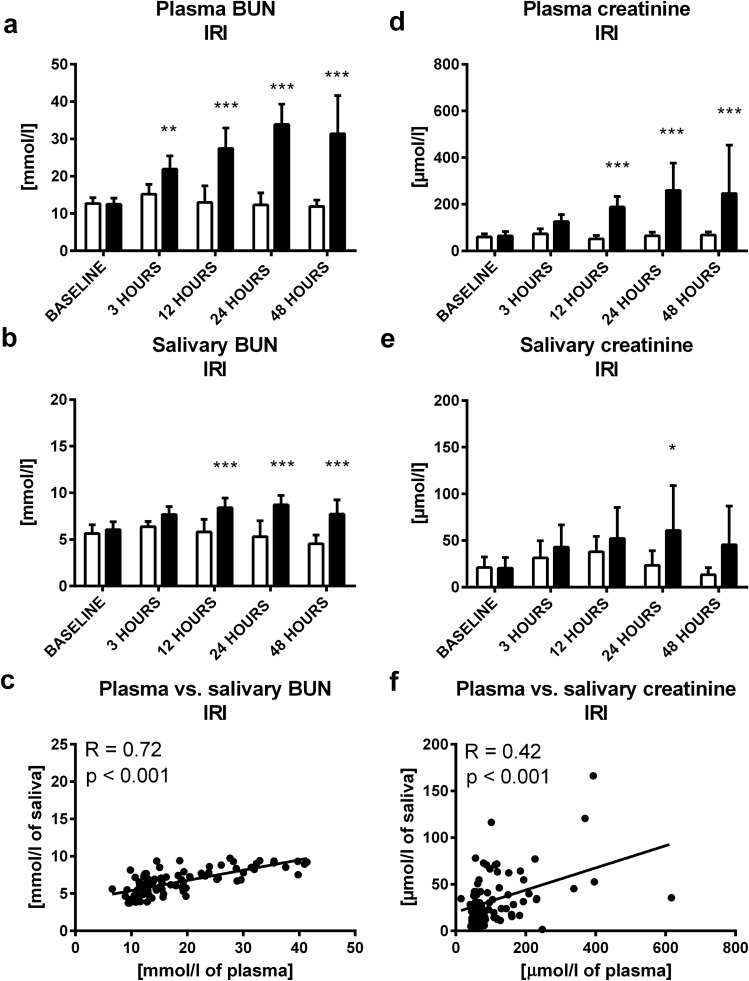
Concentration of urea in IRI or sham group in **(A)** plasma, **(B)** saliva at baseline, 3, 12, 24 and 48 h after ischemia–reperfusion injury, **(C)** correlation between plasma urea and its salivary concentrations. Concentrations of creatinine in IRI or sham group in **(D)** plasma, **(E)** saliva at baseline, 3, 12, 24 and 48 h after ischemia–reperfusion injury, **(F)** correlation between plasma creatinine and its salivary concentrations. Sham group is shown as white bars and IRI group as black bars. Results are expressed as mean + SD. * denotes p˂0.05, ** denotes p˂0.01, *** denotes p ˂ 0.001 in comparison to the sham group (n = 11 for IRI group, n = 9 for control group).

### Statistical analysis

G*Power 3.1.9.4 (Universität Kiel, Germany) was used for power analysis and determination of required sample size. Given alpha = 0.05, power = 0.80, d = 0.82 for salivary urea and d = 0.72 for salivary creatinine, the total sample size calculated was 50 for salivary urea and 62 for salivary creatinine respectively. GraphPad Prism 6.01 (GraphPad Software Inc, La Jolla, USA) was used for statistical analysis. All data were tested for normality using D’Agostino Pearson omnibus normality test. To get normal distribution of data that were not normally distributed, plasma concentrations of creatinine and salivary concentrations of urea and creatinine were log-transformed. To compare concentrations of urea and creatinine between healthy participants and patients with kidney disease, Student’s t-test was used. To compare concentrations of urea and creatinine in different stages of AKI, the Kruskal–Wallis test and subsequently Dunn’s multiple comparison test were used. To compare concentrations of urea and creatinine within stages of CKD, one-way ANOVA and subsequently Dunnett’s multiple comparison test were used. To compare concentrations of urea and creatinine in animal models, two-way ANOVA was used (one factor was time, the second factor was the treatment group) with subsequent Sidak's post-hoc multiple comparison tests. Pearson’s correlation analysis was used to evaluate differences between plasma and salivary concentrations in both, animal models and clinical samples. All values are expressed as mean + /− SD. P values below 0.05 were considered as statistically significant.

### Ethical approval

Animal experiments were approved by the Ethics Committee of the Institute of Pathophysiology, Faculty of Medicine, Comenius University, Bratislava, Slovakia. Clinical part of the study was conducted according to the principles expressed in the Declaration of Helsinki and according to approvals of the Ethics Committee of the National Institute of Children’s Diseases at the Faculty of Medicine, Comenius University, Bratislava.

### Informed consent

Informed consent was obtained from legal guardians of all participants.

## Results

### Animal experiments

In bilateral nephrectomy (BNX), plasma urea and creatinine were 40% higher than in sham controls after 3 h (t = 3.66; p < 0.01; Fig. 1A) and 180% higher after 12 h (t = 6.61; p < 0.001; Fig. 1D), respectively. Similarly, salivary urea and creatinine concentrations increased (salivary urea more than twofold after 12 h; t = 9.96; p < 0.001; Fig. 1B, salivary creatinine fourfold after 24 h since BNX (t = 3.60; p < 0.01; Fig. 1E). Both, salivary urea (R = 0.92; p < 0.001; Fig. 1C) and creatinine (R = 0.72; p < 0.001; Fig. 1F) correlated positively with the plasma concentrations.

In ischemia–reperfusion injury (IRI), plasma urea was significantly higher in comparison to sham controls 3 h after induction of IRI (t = 3.51; p < 0.01; Fig. 2A). Plasma creatinine was higher by 200% 12 h after IRI induction (t = 4.44; p < 0.001; Fig. 2D). In saliva, urea was significantly higher (by 50%) in comparison to controls 12 h after IRI induction (t = 4.99; p < 0.001; Fig. 2B), salivary creatinine was twofold higher after 24 h (t = 3.01; p < 0.05; Fig. 2E). Plasma and salivary urea (R = 0.72; p < 0.001; Fig. 2C) and creatinine (R = 0.42; p < 0.001; Fig. 2F) correlated positively and significantly.

**Figure 3:**
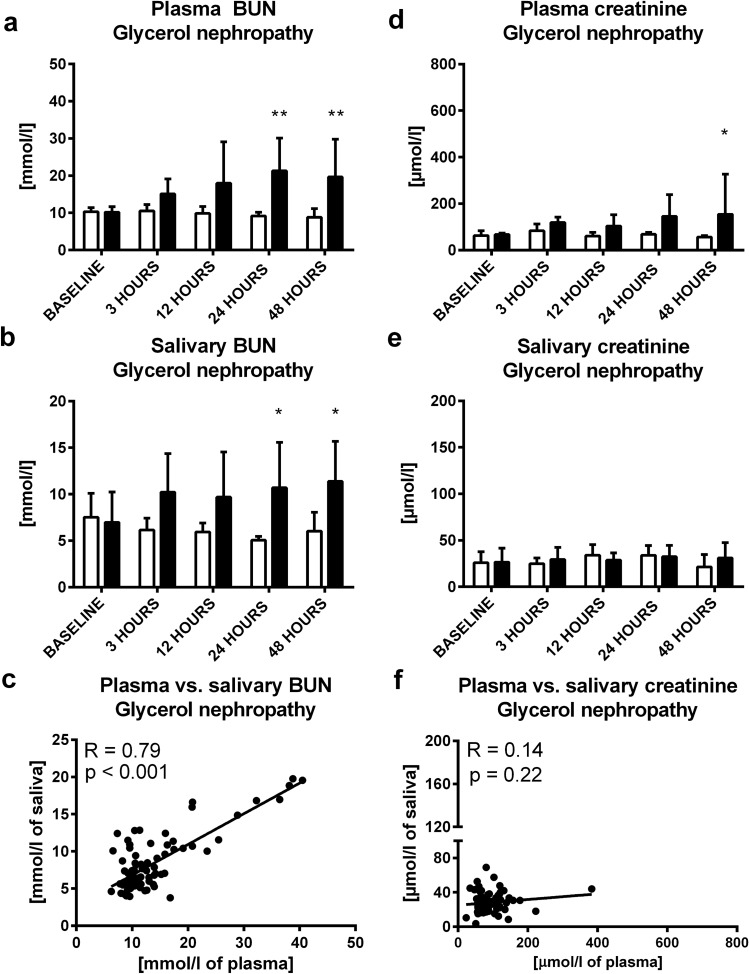
Concentration of urea in glycerol or control group in **(A)** plasma, **(B)** saliva at baseline, 3, 12, 24 and 48 h after glycerol administration, **(C)** correlation between plasma urea and its salivary concentrations. Concentrations of creatinine in glycerol or control group in **(D)** plasma, **(E)** saliva at baseline, 3, 12, 24 and 48 h after glycerol administration, F: correlation between plasma creatinine and its salivary concentrations. Control group is shown as white bars and glycerol group as black bars. Results are expressed as mean + SD. * denotes p ˂ 0.05, ** denotes p ˂ 0.01 in comparison to the control group (n = 11 for IRI group, n = 9 for control group).

In glycerol induced nephropathy, both, plasma and salivary urea were approximately twice as high 24 h after induction of the model (t = 3.55; p < 0.01 and t = 3.08; p < 0.05; Fig. 3A,B; respectively). Plasma creatinine was significantly higher in comparison to sham controls after 48 h (t = 2.67; p < 0.05; Fig. 3D). Salivary creatinine did not increase after induction of glycerol nephropathy (t = 1.49; p > 0.05; Fig. 3E). A significant and positive correlation was found between plasma and salivary urea (R = 0.79; p < 0.001; Fig. 3C), but not between plasma and salivary creatinine (R = 0.14; p > 0.05; Fig. 3F).

**Figure 4:**
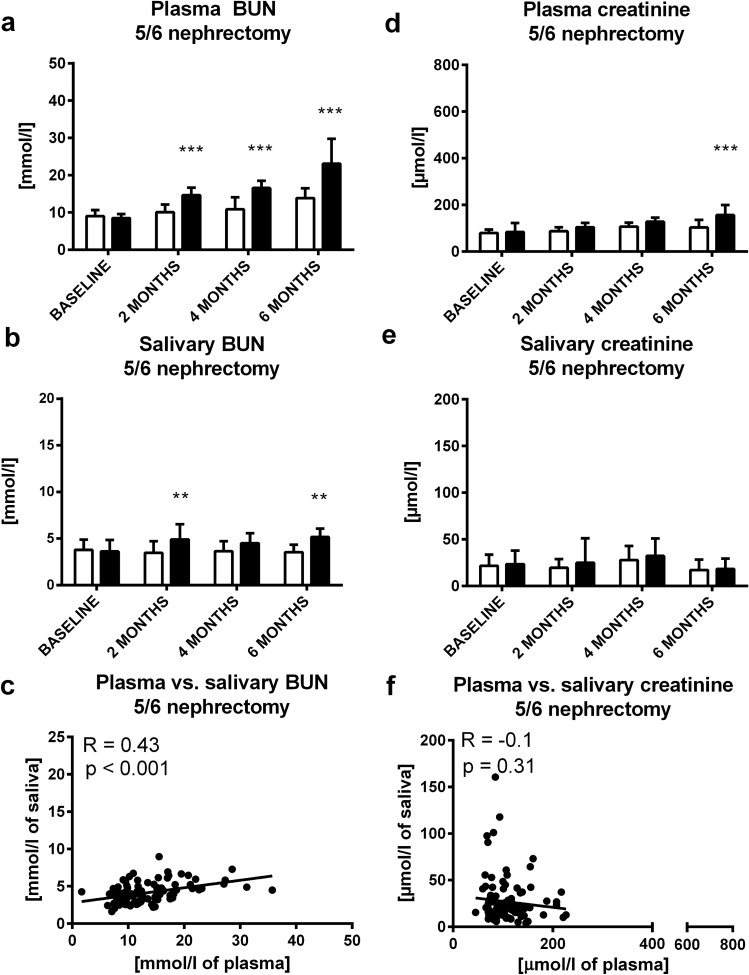
Concentration of urea in group with 5/6 nephrectomy or sham group in **(A)** plasma, **(B)** saliva at baseline, 2, 4 and 6 months after 5/6 nephrectomy, **(C)** correlation between plasma urea and its salivary concentrations. Concentrations of creatinine in group with 5/6 nephrectomy or sham group in **(D)** plasma, **(E)** saliva at baseline, 2, 4 and 6 months after 5/6 nephrectomy, **(F)** correlation between plasma creatinine and its salivary concentrations. Sham group is shown as white bars and 5/6 nephrectomy group as black bars. Results are expressed as mean + SD. ** denotes p ˂ 0.01, *** denotes p ˂ 0.001 in comparison to sham group (n = 17 for 5/6 nephrectomy group, n = 13 for control group).

In 5/6 nephrectomy as a model of CKD, plasma urea increased by 50% in comparison to the sham group after 2 months (t = 3.83; p < 0.001; Fig. 4A). This rise continued during the observed period of 6 months (t = 6.93; p < 0.001; Fig. 4A). Plasma creatinine was higher by 60% compared to sham group after 6 months (t = 4.56; p < 0.001; Fig. 4D). Salivary urea was higher by 50% after 2 and after 6 months compared to corresponding control groups (t = 3.19; t = 3.45; p < 0.01; Fig. 4B). On the other hand, 5/6 nephrectomy did not induce any changes in the salivary creatinine (t = 0.16; p˃0.05; Fig. 4E). Plasma and salivary urea correlated significantly (R = 0.43; p < 0.001; Fig. 4C), creatinine concentrations in plasma and saliva did not correlate (R = − 0.1; p˃0.05; Fig. 4F).

Although the correlations between plasma and salivary BUN and creatinine in all acute models were significant, the subgroup analysis after splitting into healthy animals and kidney injury groups revealed that the lower plasma concentrations of both markers in healthy animals did not correlate (R = 0.17, p > 0.05 for BUN and R = 0.07, p > 0.05 for creatinine). There seemed to be a threshold concentration to be reached in plasma for both markers; 11 mmol/l for BUN (R = 0.64, p < 0.001) and 160 µmol/l for creatinine (R = 0.51, p < 0.001) as determined by our experiments.

### Clinical study

**Figure 5:**
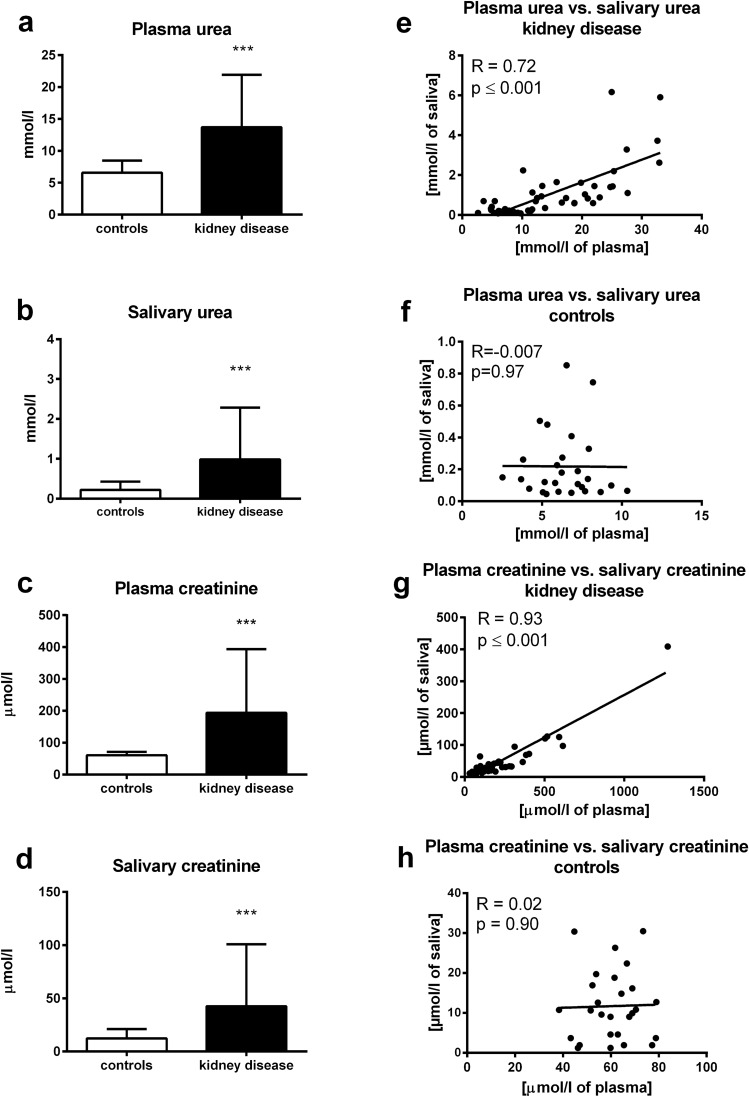
Concentrations of urea in children with kidney disease or healthy controls in **(A)** plasma, **(B)** saliva. Concentrations of creatinine in children with kidney disease or healthy controls in **(C)** plasma, **(D)** saliva. Correlations between plasma urea and its salivary concentrations in **(E)** children with kidney disease, **(F)** healthy controls. Correlations between plasma creatinine and its salivary concentrations in **(G)** children with kidney disease, **(H)** healthy controls. Results are expressed as mean + SD. *** denotes p ˂ 0.001 in comparison to healthy controls (n = 35 for children with kidney disease, n = 29 for healthy controls).

Both, plasma urea and creatinine concentrations were significantly higher in children with kidney diseases regardless of stage in comparison to healthy controls (t = 4.58; p < 0.001 for urea; Fig. 5A; t = 5.97; p < 0.001 for creatinine; Fig. 5C). Similarly, salivary concentrations of urea and creatinine were threefold higher in the group with AKI or CKD compared to healthy controls, (salivary urea – t = 4.91; p < 0.001; Fig. 5B; salivary creatinine – t = 6.38; p < 0.001; Fig. 5D). Significant positive correlations between plasma and salivary urea and creatinine were found in patients with kidney disease (R = 0.72; p < 0.001 for urea and R = 0.93; p < 0.001 for creatinine; Fig. 5E,G; respectively), but not in healthy controls (R = − 0.007; p = 0.97 for urea and R = 0.02; p = 0.90 for creatinine; 5F and 5H; respectively).

**Figure 6:**
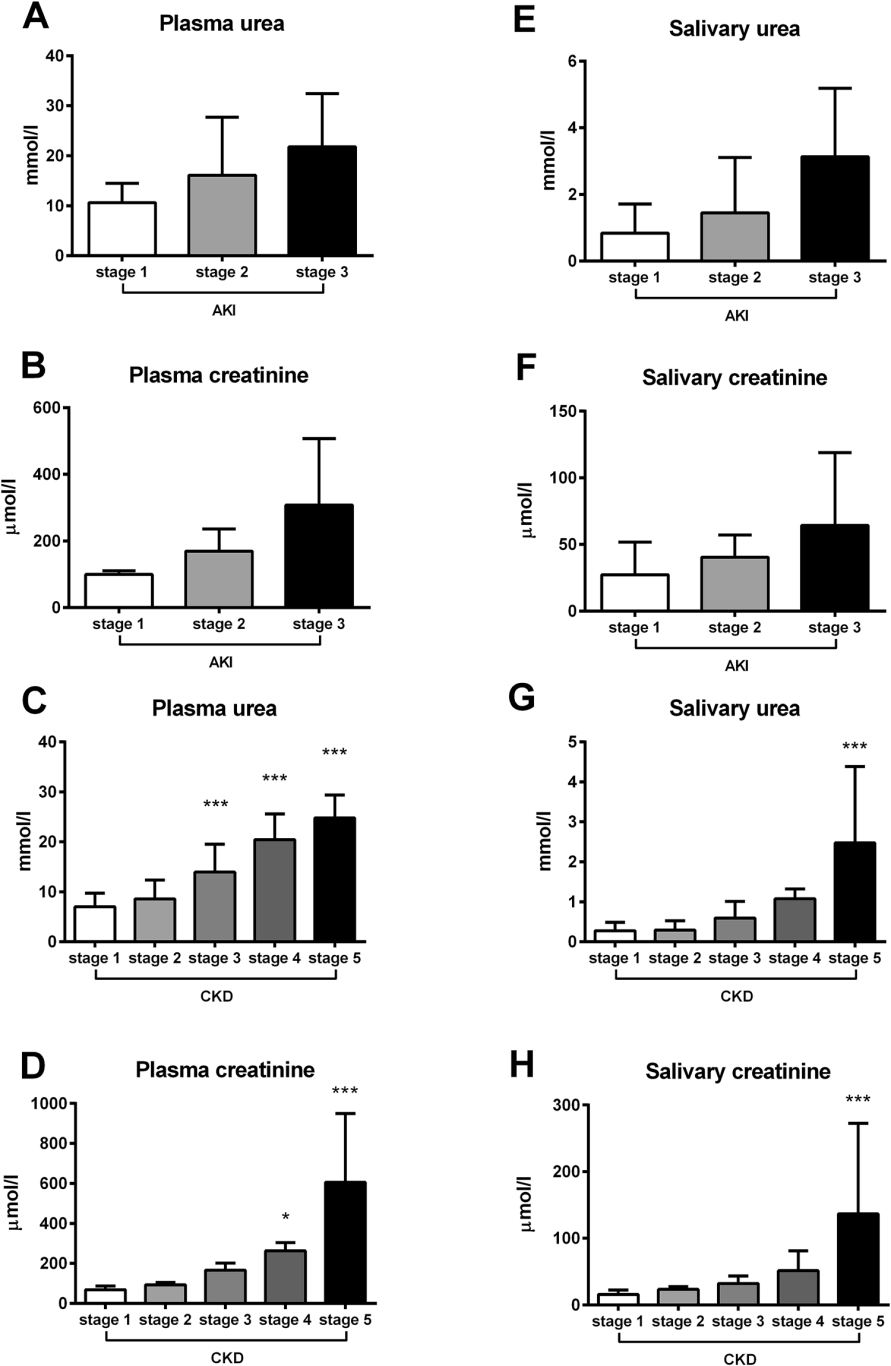
Concentrations of plasma **(A)** urea, **(B)** creatinine in different stages of AKI. Concentrations of plasma **(C)** urea, **(D)** creatinine in different stages of CKD. Concentrations of salivary **(E)** urea, **(F)** creatinine in different stages of AKI. Concentrations of salivary **(G)** urea, **(H)** creatinine in different stages of CKD. * denotes p ˂ 0.05, *** denotes p ˂ 0.001 in comparison to stage 1.

In patients with AKI, plasma and salivary concentrations of urea and creatinine increased with the stage of AKI, although the differences between the groups were not significant (Kruskal–Wallis H = 1.82; p ˃ 0.05 for plasma urea; Fig. 6A; H = 3.41; p ˃ 0.05 for plasma creatinine; Fig. 6B; H = 2.46; p ˃ 0.05 for salivary urea; Fig. 6E; H = 0.50; p ˃ 0.05 for salivary creatinine; Fig. 6F). In patients with CKD, plasma urea and creatinine increased with the stage of CKD (F = 23.68; p < 0.001 for urea; F = 22.42; p < 0.001 for creatinine). Plasma urea was significantly higher in patients with CKD stage 3 compared to CKD stage 1 (by 100%; q = 4.25; p < 0.001; Fig. 6C). Plasma creatinine was significantly higher in patients with CKD stage 4 compared to stage 1 (by 280%; q = 2.80; p < 0.05; Fig. 6D). Similarly, salivary urea and creatinine increased with the severity of CKD (F = 10.29; p < 0.001 for urea; F = 6.32; p < 0.001 for creatinine). Patients in CKD stage 4 had concentrations of salivary urea on average higher by 280% in comparison to stage 1, although this difference was not significant (q = 1.86; p ˃ 0.05). Significantly higher concentrations of salivary urea were found only in patients with CKD stage 5 in comparison to stage 1 (eightfold; q = 5.94; p < 0.001; Fig. 6G). Similarly, salivary creatinine concentrations in patients with CKD stage 4 were on average threefold higher in comparison to stage 1, but the difference was not significant (q = 1.21; p ˃ 0.05). Patients with CKD stage 5 had significantly higher concentrations of salivary creatinine in comparison to stage 1 (eightfold; q = 4.77; p < 0.001; Fig. 6H).

The post-hoc calculations of individual stages of AKI revealed the achieved power 0.27 for salivary urea and 0.19 for salivary creatinine. In CKD, the achieved power for salivary urea was 0.99 and 0.98 for salivary creatinine when adjusted to analysis of individual stages of CKD.

## Discussion

To our knowledge, this is the first study describing the detailed dynamics of concentrations of urea and creatinine in saliva during the development of AKI and CKD in various animal models and assessing concentrations of these renal markers in pediatric patients in different stages of AKI and CKD. In the animal experiments, we have analysed in detail the increase of salivary urea and creatinine during the development of AKI and CKD. The rate and extent of the increase of the salivary markers depended on the severity of renal failure. However, despite subtle differences between the models and between the markers, both salivary biomarkers mirror the increase of plasma concentrations with a delay. This could be associated with the observation that urea and creatinine increase in saliva only when plasma concentrations pass a threshold—approximately 11 mmol/l for urea and 160 µmol/l for creatinine. High plasma concentrations are likely needed for the concentration gradient that allows the diffusion of markers from blood to saliva. In the CKD model of 5/6 nephrectomy, plasma creatinine was higher than in control animals, but lower than in the AKI models. As the suggested threshold for plasma creatinine was achieved after 6 months only by few animals, this could explain why salivary creatinine did not significantly increase in this model. In contrast, plasma urea increased above threshold in rats with 5/6 nephrectomy. This is in line with our previously published animal experiments on salivary markers of renal function^17^. Previously, we showed that although plasma creatinine and urea were higher in mice with AKI and CKD in comparison to corresponding controls, their salivary concentrations were higher only in mice with AKI, but not in CKD. We assumed that an increase of salivary markers of kidney functions depends on their rise in plasma i.e. on severity of kidney disease^17^. However, in that study, blood and saliva were collected only in a single time point. To verify our assumptions, dynamics of salivary markers during the development of kidney disease was needed to monitor. Thus, our current study was an important follow up of our previous study. Our results support our hypothesis that both, plasma urea and creatinine need to reach a certain threshold to exhibit changes in saliva.

In the clinical study, we confirmed higher concentrations of urea and creatinine in saliva of children with AKI and CKD in comparison to healthy controls, which is in line with previously published studies^8,9,12^. Only few studies analysed differences in salivary urea and creatinine between stages of renal failure^10,11,26^. In our study, salivary markers of renal function increased with higher stages of AKI and CKD. However, significant differences in comparison to stage 1 were found only in children with CKD stage 5. The details of sampling and processing of saliva could be the reasons for these findings^27^. In salivary analyses bias might come from eating, drinking or toothbrushing, but these were excluded in our study. Substances that remain from the food, beverages or blood contamination of saliva might interfere with methods used for the assessment of salivary creatinine and urea. Changes of oral microbiome might influence concentrations of salivary markers due to enzymes produced by oral bacteria. Moreover, changes in salivary flow rate might result in changes of concentrations of salivary markers^6,28^. Some sources of bias might be minimized by using protocol for proper saliva collection, processing and storage of samples. However, most of them need to be identified and quantified in further studies. One potential way how to decrease the variability would be the use of a normalization factor such as urinary creatinine for urinary markers. Nevertheless, a similar factor for saliva is not yet established and is currently being discussed in the literature.

In direct comparison, salivary urea seems to be a more sensitive biomarker of renal dysfunction than creatinine in both, patients and animal models of AKI and CKD. The concentrations of urea in saliva increased sooner than those of creatinine during the development of renal failure. Our previous research has shown that while being more sensitive, salivary urea is less specific and might be affected by other factors than kidney function, for example by the periodontal status^28^. Salivary urea dipsticks and a point of care urea sensor are already available and have been tested in animals as well as in humans^29–32^. These technical improvements could be used in the monitoring of renal functions or screening of advanced CKD in veterinary medicine, but also in clinical nephrology, at least in developing countries.

A limitation of our study is the subgroup analysis, with relatively low number of patients that decreases the statistical power. Initially, the number of participants was calculated to 62 patients in order to safely observe the differences between control and kidney injury patients with enough statistical power. Although overall 86 participants were included in the study, the subgroup analysis for individual stages of AKI or CKD found reduced obtained statistical power of the analysis (AKI stages – 0.27 for BUN and 0.19 for creatinine; CKD stages – 0.99 for BUN and 0.98 for creatinine). Nevertheless, both the study and the experiment showed the salivary creatinine and BUN to be useful markers of kidney damage once the plasma thresholds are reached. It is worthy to note, that in 5/6 nephrectomy animals did not reach the plasma threshold of 160 µmol/l for creatinine or 11 mmol/l for BUN after six months. On the other hand, this was the opposite in children. Moreover, children are also likely those who would benefit the most from this approach, once salivary-based diagnostics is established in nephrology due to stress-free sample collection^33,34^. Long-term cohort observations would be needed to assess the prognostic value of creatinine or urea dynamics in saliva.

A conceptual shortcoming is the focus on urea and creatinine, which are far from being ideal biomarkers even if measured in blood plasma^35,36^. Novel biomarkers of kidney function or injury are being investigated, but an optimized protocol for the collection and processing of saliva samples as well as analysis of salivary concentrations is lacking. A multi-omics approach without a priori hypotheses would likely be more appropriate^37^ and will be used in a follow-up study. Last but not least, glomerular filtration is not the only function of the kidney. Creatinine or urea cannot be used for the assessment of any of the other endocrine or metabolic functions.

This study is the first to observe the dynamics of urea and creatinine in plasma as well as in saliva in animal models of AKI and CKD. Salivary markers of renal function increase during the development of renal failure, but later than in plasma. This is in line with the clinical data showing higher salivary urea and creatinine only in patients with the highest stages of AKI or CKD. In summary, we have shown that salivary urea and creatinine are potential biomarkers of renal function, usable for non-invasive monitoring of higher stages of AKI and CKD, but not for screening of early stages of renal diseases.

## Supplementary information

Supplementary Information.
