# Supplementary material for: Dynamics of salivary markers of kidney functions in acute and chronic kidney diseases
Source: Sci Rep. 2020 Dec 4;10:21260. doi: 10.1038/s41598-020-78209-1 (PMC7719178; doi:10.1038/s41598-020-78209-1)
Supplement: Supplementary file 1 — Supplementary Information. [file 41598_2020_78209_MOESM1_ESM.doc]

**Title:**

Dynamics of salivary markers of kidney functions in acute and chronic kidney diseases

**Authors:**

Alexandra Gaál Kovalčíková (1, 2), Kristína Ploth (1), Róbert Lipták (1), Marianna Hladová (1), Emese Renczés (1), Peter Boor (3), Ľudmila Podracká (2), Katarína Šebeková (1), Július Hodosy (1), Ľubomíra Tóthová (1), Peter Celec (1, 4)

1 – Institute of Molecular Biomedicine, Faculty of Medicine, Comenius University, Bratislava, Slovakia

2 – Department of Paediatrics, National Institute of Children’s diseases and Faculty of Medicine, Comenius University in Bratislava, Bratislava, Slovakia

3 – Institute of Pathology & Department of Nephrology, University Clinic of the RWTH Aachen, Germany

4 – Institute of Pathophysiology, Faculty of Medicine, Comenius University, Bratislava, Slovakia

**Supplementary methods**

To evaluate technical, intra- and inter-individual variability of salivary urea and creatinine, saliva was collected from 7 young and healthy volunteers from our Institute (4 women, 3 men; aged 29.3 ± 3.7). Whole unstimulated saliva was collected into sterile Falcon tubes (Sarstedt, Numbrecht, Germany) at 8 am and 4 pm for 8 consecutive days. All samples were centrifuged at 1600 g for 10 minutes. Supernatant was stored at -20°C for further analysis. Salivary creatinine and urea were measured in duplicates using the same kits as described above (Creatinine Serum Low Sample Volume, Urea Nitrogen Colorimetric Detection Kit, Arbor Assays, Ann Arbor, USA). Coefficient of variation (CV) for technical, intra- and inter-individual variability was calculated (supplementary table 2).

**Supplementary Table 1**: Clinical characteristics of individuals included in the study

| Groups | Healthy controls | Patients |
| --- | --- | --- |
| Total number | 29 | 57 |
| Age | 10.2 ± 3.7 | 12.6 ± 4.9 |
| BMI | 19.4 ± 2.3 | 20.3 ± 4.3 |
| Gender (females/males) | 12/17 | 25/32 |
| Stages: |  | |
| CKD 1 |  | 15 (26.3 %) |
| CKD 2 |  | 8 (14.0 %) |
| CKD 3 |  | 13 (22.8 %) |
| CKD 4 |  | 4 (7.0 %) |
| CKD 5 |  | 6 (10.5 %) |
| AKI 1 |  | 2 (3.5 %) |
| AKI 2 |  | 4 (7.2 %) |
| AKI 3 |  | 5 (8.8 %) |
| Etiology: |  | |
|  |  | CAKUT (29.8 %) |
|  |  | Glomerular disease (24.6 %) |
|  |  | Tubulo-interstitial disease (21.1 %) |
|  |  | Autoimmune disease (12.3 %) |
|  |  | Nephrocalcinosis (3.5 %) |
|  |  | Artery hypertension (3.5 %) |
|  |  | Others (5.3 %) |

CKD – chronic kidney disease, CAKUT – [congenital anomalies of the kidney and the urinary tract, BMI –](https://www.ncbi.nlm.nih.gov/pubmed/25313840) Body mass index

**Supplementary Table 2**: CV calculated for technical, intra- and inter- individual variability

|  | Technical CV (%) | Intra-individual CV (%) | Inter-individual CV (%) | Range  (5; 95 percentile) |
| --- | --- | --- | --- | --- |
| Salivary urea  (mmol/l) | 10.2 | 44.5 | 49.2 | 0.06; 1.97 |
| Salivary creatinine  (µmol/l) | 23.2 | 51.8 | 26.9 | 2.80; 42.19 |

CV – coefficient of variation


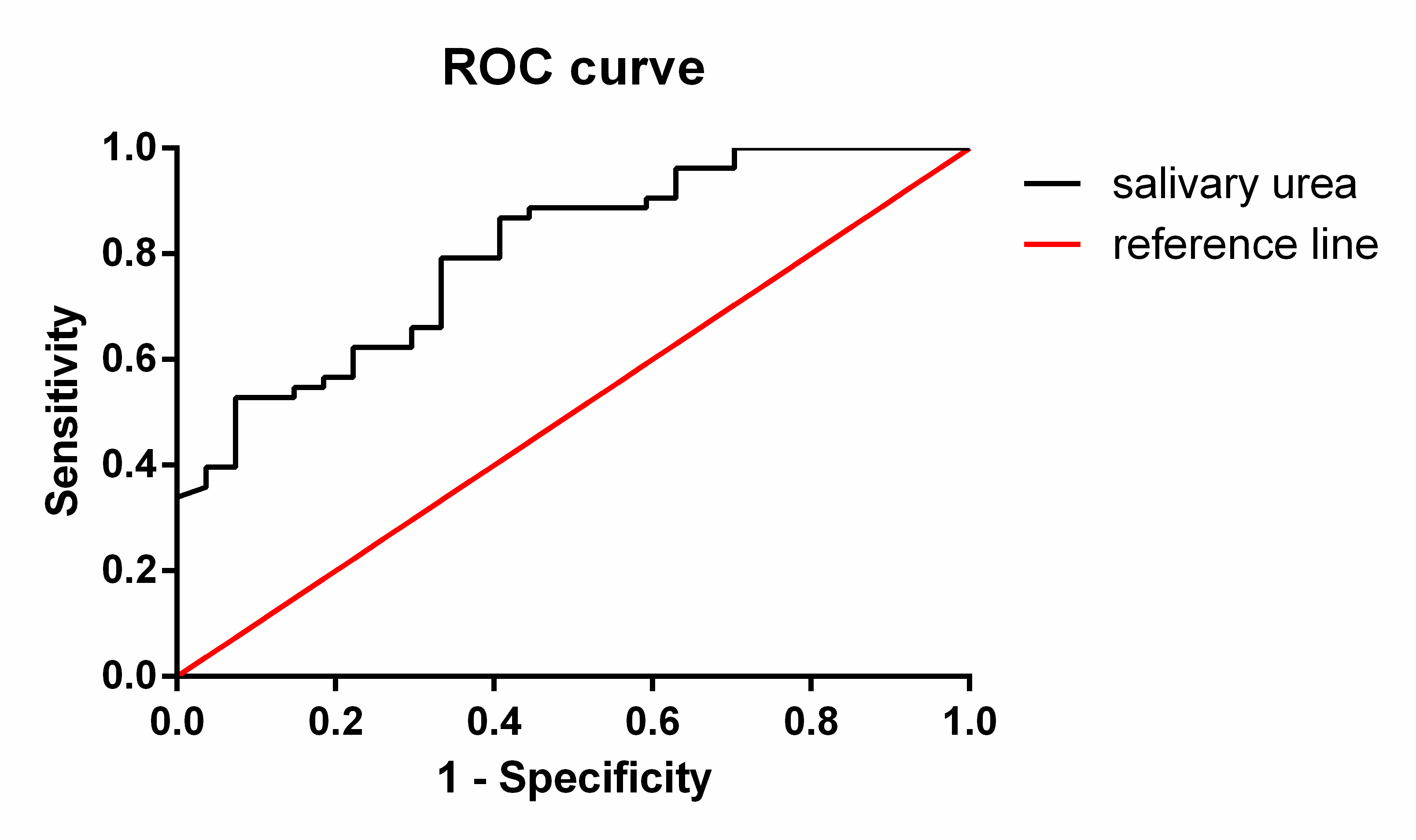


**Supplementary Fig. 1** Receiver operating characteristic (ROC) curve for salivary urea. Total area under curve is 0.80.


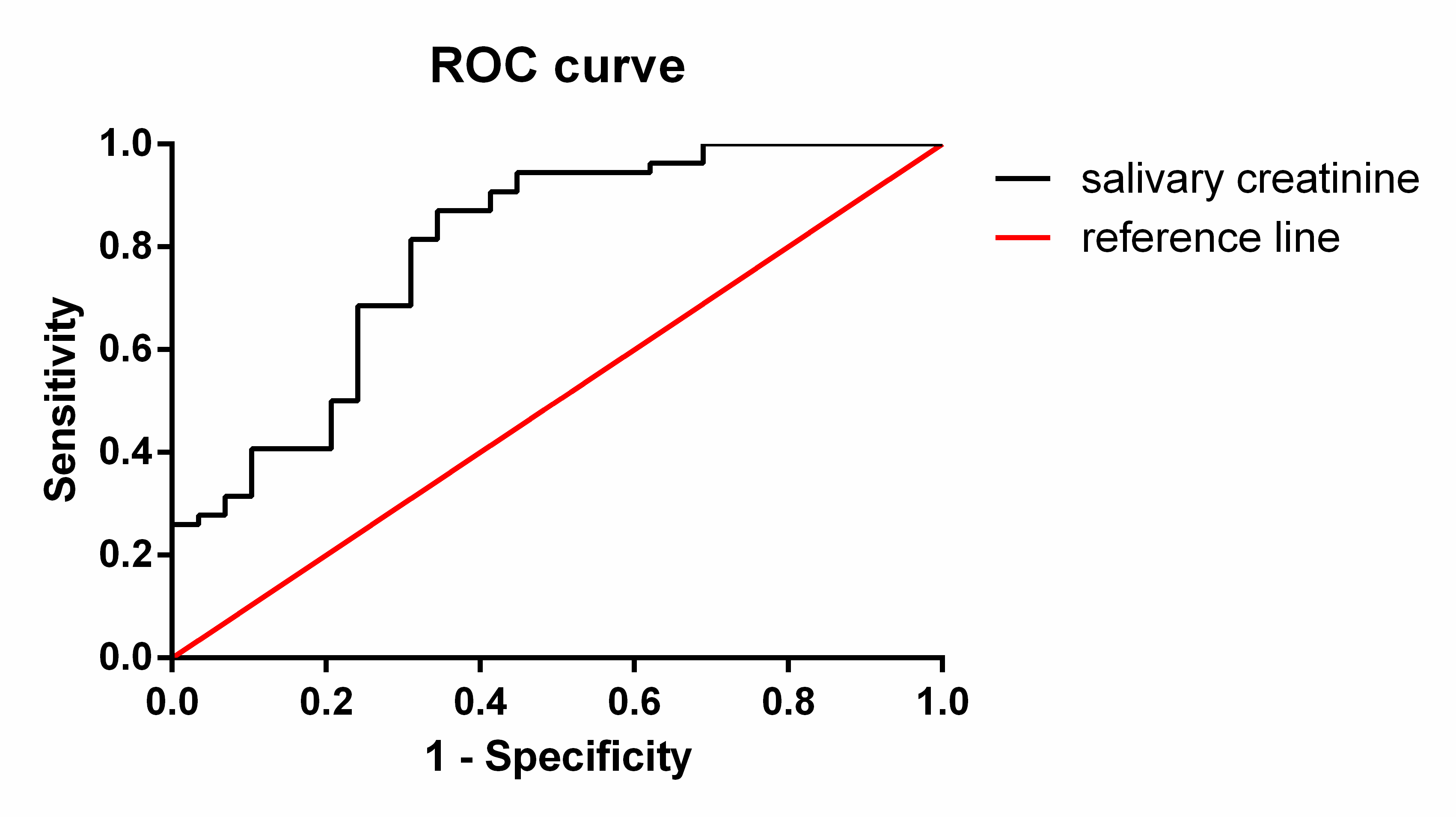


**Supplementary Fig. 2** Receiver operating characteristic (ROC) curve for salivary creatinine. Total area under curve is 0.80.
